# Long Covid requires a global response centred on equity and dialogue

**DOI:** 10.1080/16549716.2023.2244757

**Published:** 2023-08-15

**Authors:** Larry Au, Cristian Capotescu, Amanda Curi, Renan Gonçalves Leonel da Silva, Gil Eyal

**Affiliations:** a Department of Sociology, The City College of New York, New York, NY, USA; b Trust Collaboratory, Columbia University, New York, NY, USA; c Health Ethics and Policy Lab, Department of Health Sciences and Technology, ETH Zurich, Zürich, Switzerland; d Department of Sociology and Trust Collaboratory, Columbia University, New York, NY, USA

**Keywords:** Long Covid, chronic illness, Global South, patient experiences, health communication

## Abstract

Long Covid, or Post-Covid Conditions, is a global health problem. Yet we know strikingly little about the different experiences of Long Covid patients cross-nationally. To address this shortcoming, we conducted an online survey of Long Covid patients active on social media in the U.S. (*n* = 334, October to December 2021) and Brazil (*n* = 144, January to April 2022). Our analysis of short answer responses indicates patient dissatisfaction with medical care provided for Long Covid in both the U.S. and Brazil. For Long Covid patients in Brazil, there were additional concerns raised about the lack of local expertise about their condition. Based on these results, we urge policymakers to expand the education of medical professionals in order to raise awareness of Long Covid. Experts in the Global North should also be encouraged to engage in dialogue with patient groups and experts in the Global South, in order to better understand how local contexts shape the experience of Long Covid.

Long Covid, or Post-Covid Conditions, encompasses a range of experiences associated with prolonged recovery from COVID-19 [1]. The suffering caused by Long Covid is unequally distributed. *Within societies*, socio-economic inequality has exacerbated the effects of both COVID-19 and Long Covid [2]. In the United States, minority communities have been disproportionately affected owing to various factors, from workplace exposures of ‘essential workers’ to the makeup of incarcerated populations [3].

This pattern is also replicated *between countries*. While the pandemic heavily impacted both the U.S. and Brazil owing to the large numbers of individuals infected with Covid-19, its lasting effects on individuals and health systems will likely differ. In the U.S., Long Covid has received some policy attention, where patient activists successfully lobbied for National Institutes of Health funding with the RECOVER Initiative [4]. Post-Covid care centres can be found in high-income U.S. urban areas, such as at Mt. Sinai Hospital in New York City [5].

In contrast, the prioritisation of acute conditions at the expense of chronic illness is particularly pronounced in low- and middle-income countries (LMIC) [6]. This ‘double burden’ of disease is often exacerbated by the inability of LMIC healthcare systems to contend with both acute and chronic conditions [7], as well as the lack of access to medicines needed to combat chronic illnesses [8]. While Brazil’s unified health system (Sistema Único de Saúde or SUS) was designed to provide constitutionally mandated universal healthcare, the SUS has faced tremendous strain from acute COVID-19 infections during the initial phase of the pandemic [9], which compounded weaknesses inflicted by austerity measures imposed since 2016 [10]. Consequently, support for prolonged post-Covid conditions is relatively scant and patients with means often turn to private healthcare providers that fill this gap in care [11]. While the plight of Long Covid patients in the U.S. is far from ideal, Long Covid sufferers in Brazil thus have to contend with additional challenges.

As social scientists, we spent the past two years studying how Long Covid patients co-produce new knowledge to navigate uncertainties about their health condition. We launched an online survey of Long Covid patients on social media platforms in the U.S. (*n* = 334, October to December 2021) and Brazil (*n* = 144, January to April 2022) and conducted follow-up interviews with over a hundred respondents. This research was conducted by the *Covid-19 and Trust in Science Project* at Columbia University’s Trust Collaboratory. We received ethics approval from Columbia University’s Institutional Review Board (Protocol # AAAT8370) and benefited from the guidance of our Brazilian collaborators (Da Silva and Curi) as our internet-based study was only reviewed by our home institution [12].

In both our survey and interview samples, we note a bias towards females, those who were more affluent, and those who were more educated. In the Brazil sample, in turn, a larger proportion of respondents come from urban settings and identify as political independents, in contrast to more suburbanites and liberals in the U.S. sample. The characteristics of the U.S. sample can be found in Table 1, and details of the Brazil sample can be seen in Table 2. While there are limitations to this voluntary sample of social media users, our findings are nonetheless indicative of broader problems faced by Long Covid patients, particularly those who match our sample characteristics. Conceivably, the problems faced by those with lower incomes and living in more resource-poor settings will be different, and perhaps even amplified for those who are more disadvantaged. Additionally, these surveys were also conducted when the number of acute cases of COVID-19 was still somewhat high and information about Long Covid was relatively scant.

**Table 1.** Characteristics of U.S. Long Covid subsample.

Characteristic		% of Respondents
Age (Mean = 42)		
Gender	*Female*	75%
	*Male*	22%
	*Other*	3%
Race	*White*	76%
	*Black*	4%
	*Other*	20%
Education	*Graduate degree*	31%
	*Completed College*	38%
	*Some College or Below*	31%
Employment	*Full/Part Time*	54%
	*Unemployed*	6%
	*Other*	40%
Residence	*Urban*	33%
	*Suburban*	50%
	*Rural*	17%
Household Income (annual)	*$100,001 or over*	38%
	*$60,001–$100,000*	26%
	*Below $60,000*	34%
Political Identification	*Liberal/Democrat*	50%
	*Conservative/Republican*	12%
	*Independent/Other*	38%

*n* = 334, with 26 missing demographic information.

**Table 2.** Characteristics of Brazil Long Covid Subsample.

Characteristic		% of Respondents
Age (Mean = 40)		
Gender	*Female*	72%
	*Male*	28%
	*Other*	0%
Race	*White*	69%
	*Black*	13%
	*Other*	18%
Education	*Graduate degree*	28%
	*Completed College*	40%
	*Some College or Below*	32%
Employment	*Full/Part Time*	60%
	*Unemployed*	4%
	*Other*	36%
Residence	*Urban*	86%
	*Suburban*	13%
	*Rural*	1%
Household Income (monthly)	*R$*11,000 *or over*	14%
	*R$5501–10,999*	24%
	*R$1,101–5,500*	50%
	*Below R$1,100 (minimum wage)*	12%
Political Identification	*Progressive/Center-Left*	25%
	*Conservative/Center-Right*	6%
	*Independent/Other*	69%

*n* = 144, with 19 missing demographic information.

Our analysis compared the short answer responses of two surveys investigating the following issues: (1) respondents’ experiences of receiving medical treatment and (2) their perceptions of trusted sources of information in relation to Long Covid. We were struck by two findings.

First, despite the different national contexts in which the pandemic occurred, Long Covid sufferers in both countries recounted experiences of being dismissed by medical professionals. These responses were qualitatively coded as positive, negative, or neutral/unclear (positive and negative responses are not mutually exclusive). In the Brazil sample, 61% reported negative interactions and 22% reported positive interactions. In the U.S. sample, 79% reported negative interactions and 21% reported positive interactions.

When asked to describe their interactions with doctors in short-answer responses, the majority of U.S. and Brazilian respondents pointed to negative experiences. U.S. patients, in particular, mobilised the rhetoric of ‘medical gaslighting’ to accuse their medical providers of dismissing their reports of symptoms and refusing to believe that what they were experiencing could be attributed to Long Covid [13].

While this rhetoric of gaslighting is not as prominent in Brazil, many Brazilian patients also recalled being told that their symptoms were ‘just anxiety.’ As one Brazilian respondent put it, ‘Doctors who are not well informed […] tend to psychologize any and all symptoms’. Another respondent reported, ‘Doctors look at me like I’m lying.’ Brazilian respondents also noted that doctors – primary care and specialists – lacked awareness about Long Covid. As one stated: ‘Doctors don’t know what to treat. Don’t have the knowledge.’ But whereas at least some U.S. patients could turn to post-Covid care centres, where there was more acceptance and recognition of Long Covid diagnoses as well as access to specialised care, Brazilian respondents expressed additional difficulties in accessing any form of Long Covid – competent care.

Second, because of this lack of local medical expertise, Long Covid patients have turned to online patient communities, seeking advice from fellow sufferers to overcome the knowledge gap in traditional sources of medical expertise [14]. Many U.S. respondents explained this decision saying, ‘I trust other Long Covid patients the most.’

Our Brazilian respondents noted similar experiences, with one individual writing that they trust, ‘testimonials and commentaries of people that lived or are living the situation [because] they contribute details about the symptoms and information about the lack of attention from the healthcare system.’ This is not naïve trust; respondents knew the potential for misinformation and often did the added work of verifying information about treatments mentioned in online discussions. These communities also provided valuable sources of emotional support to patients.

Brazilian respondents, however, also noted that trustworthy information about Long Covid seemed to come from overseas, from experts in the U.S. or Europe. For instance, Brazilian respondents reported that they turned to ‘French YouTube channels,’ the ‘United Nations,’ ‘foreign newspapers,’ and ‘studies from the U.K.’ to seek out what they perceived to be credible information about Long Covid. These sources were chosen to supplement local news and experts and what other patients shared online.

This is understandable, given the relative dominance of U.S. and European scientists involved in Long Covid research – as well as in health research more generally [15]. A cursory search on December 2022 for the term ‘Long Covid’ at Dimensions.ai found 1297 publications receiving support from U.S. funders, while publications supported by Brazilian funders totalled 132. Of course, given that Long Covid is still a relatively new topic, these numbers may change over time. The relative scarcity of Brazil-specific research – with several important and notable exceptions [16–18]—is also likely to impact the perceived legitimacy of Long Covid in Brazil as well as the development of social support and therapies suited to the Brazilian context.

Experts in public health and medicine are starting to grapple with the long-term effects of Covid-19. We provide three recommendations for policymakers and medical providers based on our work.

First, as evidenced by U.S. and Brazilian survey responses, awareness of Long Covid remains inadequate among frontline medical providers. Pointing this out is not to place blame on medical professionals individually, but rather to point to the systemic barriers that face patients as they seek out Long Covid-competent care. Part of this has to do with the time required for medical systems and institutions, in general, to catch up with this and many other new health conditions. But more education and resources are also needed for medical professionals: Physicians, nurses, and others should be given additional training and support to recognise and validate patient experiences while delivering medical care for Long Covid patients. This will serve as an initial step to ensuring that Long Covid patients are believed, and will increase the likelihood that their medical needs will eventually be met.

Second, further dialogue with Long Covid patients is needed globally. While patient-led research in the U.S. has played a crucial role in advancing our understanding of Long Covid [19], more can be done to support the development of similar patient initiatives in places like Brazil in order that patients from these countries can consolidate their knowledge about Long Covid in accordance with their experiences. Long Covid patients have become, of necessity, experts of their own conditions and are valuable sources of knowledge for providers. Thus, policymakers should establish lines of communication with these patient groups.

Third, it is necessary to create avenues of communication between patient communities and Long Covid experts in the Global South and those in the Global North. Learning more about cross-national differences could help us better understand Long Covid better in the long run, particularly in terms of how social determinants of health shape Long Covid [20]. Promoting knowledge exchange internationally can also help create informal networks where information about new treatments can be shared. Sensitivity to local contexts of care will also enable policymakers to better design social policies such as on disability and workers’ compensation, which in turn will also do much to alleviate the burden of Long Covid patients. Extra care should also be taken to include the voices of those who are doubly disadvantaged – patients in the Global South who experience marginalisation owing to within-country inequalities in addition to the between-country differences that we have highlighted.

To truly confront Long Covid, global health must place equity front and centre by recognising global inequalities in awareness, resources, and political willpower.

## Context

To date, there have been a growing number of studies that try to characterise the epidemiology and experiences of patients suffering from Long Covid in the Global North, such as in the United States. Less is known about Long Covid globally, particularly in the Global South. Our study begins to look at the relevant points of comparison between the patient experiences with Long Covid in the United States and Brazil.
